# Glycogen Synthase Kinase-3: A Focal Point for Advancing Pathogenic Inflammation in Depression

**DOI:** 10.3390/cells10092270

**Published:** 2021-09-01

**Authors:** Ryan T. McCallum, Melissa L. Perreault

**Affiliations:** 1 Department of Biomedical Sciences, Ontario Veterinary College, University of Guelph, Guelph, ON N1G 2W1, Canada; rmccal01@uoguelph.ca; 2 Collaborative Program in Neuroscience, University of Guelph, Guelph, ON N1G 2W1, Canada

**Keywords:** MDD, depression, glycogen synthase kinase 3, inflammation, oxidative stress

## Abstract

Increasing evidence indicates that the host immune response has a monumental role in the etiology of major depressive disorder (MDD), motivating the development of the inflammatory hypothesis of depression. Central to the involvement of chronic inflammation in MDD is a wide range of signaling deficits induced by the excessive secretion of pro-inflammatory cytokines and imbalanced T cell differentiation. Such signaling deficits include the glutamatergic, cholinergic, insulin, and neurotrophin systems, which work in concert to initiate and advance the neuropathology. Fundamental to the communication between such systems is the protein kinase glycogen synthase kinase-3 (GSK-3), a multifaceted protein critically linked to the etiology of MDD and an emerging target to treat pathogenic inflammation. Here, a consolidated overview of the widespread multi-system involvement of GSK-3 in contributing to the neuropathology of MDD will be discussed, with the feed-forward mechanistic links between all major neuronal signaling pathways highlighted.

## 1. Introduction

Major depressive disorder (MDD) is a debilitating mental health condition that has an estimated lifetime prevalence of 16.2% in the American public [1]. The symptoms of MDD include a loss of pleasure, fatigue, feelings of worthlessness, and the diminished ability to think or concentrate [2,3]. MDD is the leading cause of disability worldwide and is the most prominent mental illness amongst suicidal individuals [4,5,6].

Dysfunctional monoamine neurotransmission has been a well-documented characteristic of MDD for over 50 years, with the most commonly prescribed type of antidepressants, selective serotonin reuptake inhibitors (SSRIs), owing their mechanism of action to this hypothesis [7,8]. In the half century since this hypothesis originated, there have been many discoveries regarding the etiology of MDD that have expanded upon this single serotonin (5-HT) system view. Indeed, widespread clinical and preclinical investigations have shown that MDD is an incredibly complex disorder with an inherent pathogenesis involving multiple systems critical to brain function, which include glutamatergic signaling, cholinergic signaling, neurotrophin signaling [9], insulin signaling, inflammatory signaling, and the management of oxidative stress [10,11]. Fundamental to the pathology of MDD is the interconnected nature of such systems and how they interact in states of chronic psychological strain, leading to a system-wide dysfunction. Inflammation, which can be induced by psychological stress, has been shown in recent years to host a critical regulatory role in the activity of the *N*-methyl-*D*-aspartic acid (NMDA) signaling, 5-HT synthesis and signaling, dopamine (DA) signaling, gamma-aminobutyric acid (GABA) signaling, and the increased appearance of oxidative stress, contributing to a series of downstream consequences that mirror that of MDD [12,13]. As a result of such actions, it is now largely believed that chronic inflammation plays a significant role in the presentation of MDD and may be responsible for a large subset of cases. Given the interrelated nature of such systems in the etiology of MDD, it is without surprise that many research efforts have devoted their resources into the discovery of novel therapeutics to combat one of these systematic dysfunctions. Unfortunately, as the SSRIs have demonstrated over the years, monotherapeutic strategies are largely hit or miss, with a significant time lapse before therapeutic effects emerge [14], and major subsets of the population becoming non-responsive to treatment [15]. It is therefore of utmost importance that multi-target approaches in MDD treatment are considered.

**Figure 1:**
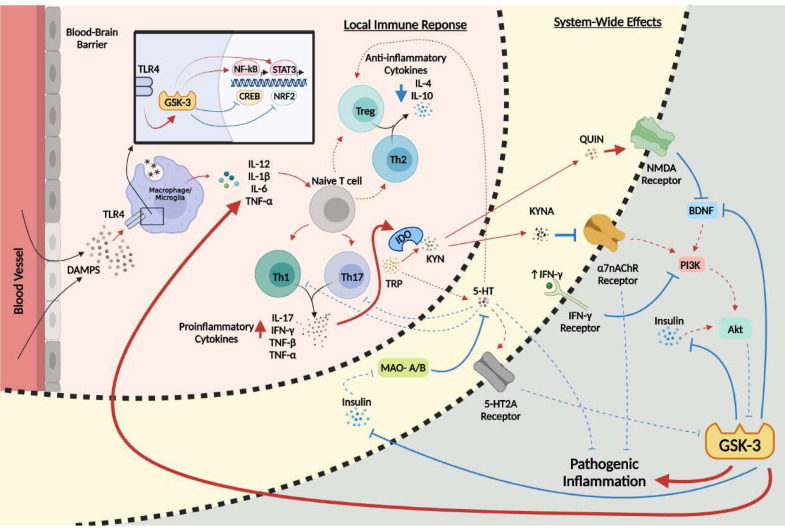
Multifaceted involvement of GSK-3 in promoting MDD neuropathology. Several bidirectional signaling pathways work cooperatively to elevate GSK-3 activity in MDD. Such processes, initiating from the DAMP-mediated activation of the TLR4, include the heightened production of inflammatory cytokines from NF-κB and STAT3 with subsequent suppression of anti-inflammatory cytokines and antioxidant factors from Nrf2 and CREB. Imbalanced cytokine production results in modified cellular differentiation of T helper cells into pro-inflammatory Th1 and Th17 cell variants, with synchronous reductions to the proliferation of Th2 cells and Tregs. Inflammatory cytokines TNF-α, IL-6, IL-1β, and IFN-γ stimulate IDO to metabolize TRP into KYN which is further metabolized into QUIN and KYNA. Downstream effects of IDO activation include reduced 5-HT and α7nAChR signaling, and enhanced NMDA and IFN-γ signaling, collectively inhibiting PI3K and Akt, and disinhibiting GSK-3, which subsequently inhibits the action of insulin. Positive regulation (red arrows) and negative regulation (blue arrows) of each pathway and/or process are shown. Changes in the overall activity of these pathways compared to normal conditions are represented by the solid arrows (increased activity) and dotted arrows (decreased activity). α7nAChR, α7 nicotinic acetylcholine receptor; BDNF, brain-derived neurotrophic factor; CREB, cAMP-response element binding protein; DAMP, danger-associated molecular patterns; GSK-3, glycogen synthase kinase 3; IDO, indoleamine 2,3-dioxygenase; IFN-γ, interferon gamma; IL-1β, interleukin 1 beta; IL-4, interleukin 4; IL-6, interleukin 6; IL-10, interleukin 10; IL-12, interleukin 12; IL-17, interleukin 17; KYN, kynurenine; KYNA, kynurenic acid; MAO-A/B, monoamine-degrading enzyme A/B; NF-κB, nuclear factor kappa B; NMDA, *N-methyl-D*-aspartic acid; NRF2, nuclear factor-erythroid factor 2-related factor 2; PI3K, phosphoinositide 3-kinase; QUIN, quinolinic acid; STAT3, signal transducer and activation of transcription 3; Th1, T helper cell type 1; Th17, T helper cell type 17; Th2, T helper cell type 2; TLR4, toll-like receptor 4; TNF-α, tumor necrosis factor alpha; TNF- β, tumor necrosis factor beta; Treg, regulatory T cell; TRP, tryptophan; 5-HT, serotonin. Figure created with BioRender.com.

At the forefront of the network of systems implicated in the pathology of MDD is the serine/threonine protein kinase glycogen synthase kinase-3 (GSK-3), a multifaceted protein with a multitude of physiological functions and over 40 known substrates that regulate gene expression, cell survival, and neuronal polarity [16]. GSK-3 exists in two isoforms, GSK-3α and GSK-3β, with the latter having extensive implications in neurodegenerative diseases such as Alzheimer’s disease [17,18,19] and neuropsychiatric disorders such as schizophrenia [20]. GSK-3 has an extremely diverse role in many cellular cascades, with numerous targets and modulators involved in essential pathways of the central nervous system (CNS), such as the immune, serotonergic, and glutamatergic systems [12,21]. Importantly, the topic of GSK-3 in the context of inflammation (reviewed in [22]), MDD (reviewed in [18]), and inflammation in MDD (reviewed in [23]) have been covered in numerous reviews; however, here we posit that GSK-3 may act as a central convergence point and important pathological hub amongst all major systems implicated in the inflammatory hypothesis of depression. Thus, the purpose of this comprehensive review is multifactorial, as it will identify how GSK-3 contributes to MDD at large, how the kinase promotes chronic low-grade inflammation, and how this inflammation then acts in combination with the other downstream targets of the kinase to promote the development of MDD (Figure 1). This review will also emphasize the fundamental need for the scientific community to investigate multi-targeted approaches for the symptomatic treatment of MDD progression.

## 2. GSK-3 in MDD

Aberrant GSK-3 activity is a well-documented hallmark of MDD, with studies explicitly demonstrating an intricate relationship between GSK-3 and depressive behavior. For example, several gene studies have associated elevated gene transcript levels of GSK-3 with MDD risk [24,25,26,27]. Oh and colleagues [25] found the post-mortem levels of GSK-3β mRNA expression in the hippocampus (HIP) of individuals with MDD to be significantly elevated compared to healthy controls. Moreover, Numata and colleagues [26] demonstrated significantly lower DNA methylation rates in patients with MDD at the CpG site in the CpG island (CGI) of the GSK-3β promoter region, with significantly higher GSK-3β mRNA expression levels in the blood of the same patients. With regard to enzymatic activity, GSK-3 activity levels are elevated in depressed individuals with elevated GSK-3 activity, but not total GSK-3 protein levels, in the post-mortem ventral prefrontal cortex (vPFC) of suicidal and non-suicidal MDD patients compared to that of healthy controls [28]. Additionally, evidence has been shown by Li et al. [29] that demonstrates increased GSK-3 activation in peripheral blood mononuclear cells, an effect ameliorated with lithium treatment. Moreover, several genes encoding proteins involved in regulating the function of GSK-3 have shown polymorphisms associated with regional gray matter volume changes in patients with MDD [29]. This included several Wnt signaling pathway genes in regions such as the right hippocampus for *rs7224837* (*Axin2*) and *rs13002663* (*Zeb2*) and the temporolateral cortex for GSK-3β substrate genes, such as *rs3785880* (*Mapt*) and *rs10482655* (*Nr3c1*) [30]. Additionally, an association with MDD has been made between the polymorphism of *rs1130214* and *rs3730358* in the *AKT1* gene, an upstream regulator of GSK-3, and in the GSK-3β gene *rs334558*, which correlated with the occurrence of the condition and rate of remission [31]. Furthermore, *rs334558*, along with *rs13321783* and *rs2319398*, additional GSK-3β single-nucleotide polymorphisms (SNPs), have also been shown to have a strong association with the 4-week SSRI antidepressant therapeutic response [21,32].

In the context of preclinical work, an increased GSK-3 function in the hippocampus has become a well-documented characteristic in popular models of depression, such as the chronic mild stress (CMS) and unpredictable alternating ultrasound models [33,34]. In addition, using a mutant tryptophan hydroxylase 2 knockin mouse model that exhibited a reduced 5-HT synthesis by roughly 80 percent, Beaulieu and colleagues [35] observed marked increases in GSK-3β activation in the frontal cortex, along with stereotypical behaviors, which were effects that were normalized by the GSK-3 inhibitor 4-benzyl-2-methyl-1,2,4-thiadiazolidine-3,5-dione (TDZD-8). Studies on mice have also effectively demonstrated the inhibition of GSK-3 to be anti-depressant using the GSK-3 inhibitors VP2.51 and thiadiazolidinone NP031115, further illustrating the potential involvement of GSK-3 in the etiology of MDD [36,37]. Furthermore, Omata and colleagues [38] discovered immobility times in both the forced swim test (FST) and tail suspension test (TST), which are tests of depression-like behavior, to be reduced following GSK-3β inactivation via intra-hippocampal injections of lentivirus-expressing short-hairpin RNA targeting GSK-3β. In contrast, increased GSK-3 activity in knockin mice has been associated with an increased susceptibility to learned helplessness and immobility time during these same tests [39]. Interestingly, the popular antidepressant fluoxetine has had its therapeutic effects recently linked to the activity of GSK-3 in E15–16 Wistar rats, as it has been shown to upregulate the kinase Akt, an upstream modulator of GSK-3, inhibiting its activity via the phosphorylation of ser9/21 [40]. Importantly, the downregulation of Akt enzymatic activity has been demonstrated in the ventral PFC of depressed suicide victims [41], with the activation of the kinase in the PFC of rats, via ketamine administration, eliciting anti-depressant effects [42]. In summary, GSK-3 has been shown to have a strong link to MDD pathogenesis, with its upregulation observed in both preclinical and clinical models of the disorder. With respect to downstream mechanistic actions of GSK-3 in MDD, the protein is known to disrupt a plethora of critical systems, such as those involved in inflammatory activation, 5-HT synthesis, cholinergic signaling, and glutamatergic function.

## 3. GSK-3 and Inflammation

The adaptability of living organisms to interpret and respond to their external environment is largely mediated by the host immune system through diverse signaling pathways that result in the presentation of inflammation and oxidative stress (reviewed in [22,43]). Subdivided into two main lines of deterrence, the fast-acting innate immune system carries out the organism’s initial line of defense by utilizing cells such as macrophages and dendritic cells to engulf potential microbial pathogens, with the subsequent presentation of corresponding antigens to the slow-acting adaptive immune system activating and producing B lymphocytes (B cells) and T lymphocytes (T cells) [22,43,44].

Fundamental to the function and action of both immune subsystems is the production and systematic circulation of the signaling molecules known collectively as cytokines [22,43,44]. Falling into categories of pro-inflammatory and anti-inflammatory, these small, physiologically essential proteins mediate the activity of many immune cell populations, including the modulatory T helper cells, type 1 T helper cell (Th1), Th2, and both Th17 and the specialized subpopulation of T lymphocytes known as regulatory T cells (Tregs) [22,43,44,45]. The cytokines responsible for pro-inflammatory states include, but are not limited to, interleukin 1β (IL-1β), tumor necrosis factor alpha (TNF-α), IL-6, IL-12, and interferon gamma (IFN-γ), with anti-inflammatory cytokines consisting of IL-4, IL-6, IL-10, IL-11, and IL-13 (reviewed in [43,45,46,47]). In recent years, GSK-3 has received increasing attention as a promising therapeutic for inflammatory disease due to its well-documented role in regulating both the innate and adaptive immune systems, and promoting cytokine production through altered T helper cell differentiation [22,43,48,49,50,51,52,53].

### 3.1. Immune Regulation of GSK-3

In order for the host immune system to accurately combat stress, trauma, or infection, a population of transmembrane receptors known collectively as Toll-like receptors (TLRs) must be activated in order to initiate a response [54,55]. In the human body, there are ten distinct TLRs that help immune cells to accurately distinguish between the self and non-self via a wide variety of ligands that correspond to each unique subtype [56]. Of the various TLRs documented in humans and rodents, the Toll-like receptor 4 (TLR4) has shown the ability to stimulate GSK-3 activity and promote pro-inflammatory cytokine release [43,57,58,59]. TLR4 activation operates through the binding of both endogenous ligands, such as danger-associated molecular patterns (DAMPs), also known as alarmins, or exogenous ligands, such as pathogen-associated molecular patterns (PAMPs) and lipopolysaccharides (LPS) [43,60,61,62]. During prolonged states of psychological stress, endogenous levels of DAMPs increase and trigger an inflammatory response via the TLR4 receptor, which both utilizes and promotes the activity of GSK-3 [43,57,58,61,62,63]. This pathway collectively inhibits anti-inflammatory cytokine release and promotes pro-inflammatory cytokine production in a number of immune cell types [22,43,44,57,58]. The link between the TLR4 and GSK-3 has been firmly established through the use of TLR4 knockout mice in both a model of learned helplessness [58] and ischemia [63], with the absence of the receptor significantly reducing GSK-3 activity, which correlates with reductions in cytokine accumulation [58,63]. In addition to the DAMP-mediated activation of TLR4, physiological stress can induce the activation of GSK-3 via the steroid hormone cortisol, or corticosterone in rodents; however, the exact mechanism behind such GSK-3 activation is largely unknown [43,64].

### 3.2. GSK-3 Regulates Immune Cell Differentiation

Cellular differentiation is a fundamental component of the adaptive immune system’s strategy against endogenous and exogenous insult [22,44,65]. This process consists of naïve CD4+ T cells developing into functional T helper cells, such as Th1, Th2, Th17 and Tregs, that function to produce distinct cytokines corresponding to their specific roles [22,44,65]. Importantly, GSK-3 can manipulate immune cell differentiation, favoring those subtypes such as Th1 and Th17 that promote pathogenic inflammation [50,66,67,68,69].

#### 3.2.1. Th1 Cells

The Th1 cell is a pro-inflammatory adaptive immune cell responsible for the production of the cytokines IFN-γ, IL-2, TNF-α, and TNF-β, and the activation of macrophages [46,47,65]. The differentiation of a Th1 cell from a naïve T cell is largely governed by extracellular concentrations of IL-12, which act to increase Th1 populations [46,47,65]. In conjunction with its capacity to promote the proliferation of a variety of pro-inflammatory cytokines, GSK-3 also has the capability to control Th1 differentiation via IL-12 [44,50,70]. Notably, Beurel et al. [50] demonstrated the marked reduction in Th1 cell proliferation in male C57BL/6 and female SJL/J mice following the localized inhibition of GSK-3 in the spinal cord. Additional reports have further linked the proliferation of Th1 cells to the activity of GSK-3 by GSK-3α knockdown in mice [50], as well as with the manipulation of GSK-3 regulators, such as phosphoinositide 3-kinase (PI3K), and the downstream targets of the kinase, including nuclear factor kappa B (NF-κB) and the signal transducer and activator of transcription 3 (STAT3) [50,66,71,72].

#### 3.2.2. Th2 Cells

The Th2 cell is another mediator of pathogenic inflammation in the host immune system, responsible for the regulation of cytokines such as IL-4, IL-5, IL-10, and IL-13 [46,47]. This helper T cell variant inhibits several macrophagic functions and is largely anti-inflammatory by nature [46,47]. Differentiation from a CD4+ T cell into a Th2 is induced by IL-4 and inhibited by IL-12, respectively [47,65]. Unlike that of the Th1 cell, GSK-3 and its downstream targets act to moderately inhibit Th2 proliferation via both the downregulation of cytokines such as IL-4 that are responsible for Th2 differentiation, and the upregulation of IL-12 [50,71,72].

#### 3.2.3. Th17 Cells

The Th17 T helper cell variant is a producer of the highly inflammatory cytokines IL-17, TNF-α, and IFN-γ, amongst many others (reviewed in [73]). Th17 cells are differentiated from naïve T cells through a combination of transforming growth factor beta (TGF-β) and IL-6 exposure, with TNF-α and IL-1β further enhancing Th17 differentiation [73]. Importantly, both Th17 proliferation and its hallmark cytokine IL-17 have been definitively linked to GSK-3 in a mutually beneficial manner, with each protein demonstrating the ability to promote the action of the other [50,66]. Several studies by Beurel et al. [50,66] have investigated the effects of GSK-3 inhibition on Th17 activity and have demonstrated that both inhibition and protein knockout strongly diminish the functionality of Th17 and its rate of proliferation in mice. Interestingly, this group also found that the direct activation of GSK-3 and its downstream targets, such as NF-κB and STAT3, also increases Th17 levels [66]. Of importance, the anti-inflammatory cytokine IL-4, as well as IL-10, which is regulated by Treg cells, both decrease Th17 proliferation, providing a regulatory role for the Th2 and Treg cells in preventing pathogenic IL-17 release [65,74].

#### 3.2.4. Treg Cells

In addition to the T helper cell variants, naïve T cells have the capability to differentiate into regulatory Tregs, which act as inflammatory modulators to the host immune system [75,76]. These regulatory cells produce anti-inflammatory cytokines, such as IL-10 and TGF-β, in order to prevent chronic inflammation and system-wide stress [65,75,76]. In contrast to the GSK-3-induced activation of Th1 and Th17, Tregs are largely inhibited by the kinase, as shown by Graham and colleagues [67], who reported that inhibition of the GSK-3β enhanced activation of Tregs in mice suppressed inflammation. The mechanism of action relied significantly on β-catenin, with a higher activity of the kinase resulting in the downregulation of both β-catenin and the corresponding Tregs [67,68].

#### 3.2.5. Innate Immune Cell Types

The innate immune system comprises numerous cell types that receive modulation from GSK-3 in several pathways, such as cell survivability and cytokine production [77,78]. Such GSK-3-regulated cells include, but are not limited to, macrophages, monocytes, natural killer cells, microglia, and dendritic cells [77,78]. Yuskaitis and Jope [49] found that the treatment of BV-2 microglia with GSK-3 inhibitors greatly reduced the migration of microglia in both a scratch assay and in a transwell migration assay. Furthermore, they found the cytokine production, microglial migration, and inflammation-induced neuronal toxicity in the hippocampus of C57BL/6 mice to be reduced following GSK-3 inhibitor treatment [49]. Rodionova et al. [79] reported that GSK-3 had the ability to inhibit macrophage development during differentiation and suppressed the IL-4/granulocyte–macrophage colony-stimulating factor (GM-CSF)-mediated differentiation of human monocytes into dendritic cells.

### 3.3. GSK-3 Modulates Inflammatory Regulators

The host immune system utilizes GSK-3 in several regulatory pathways in order to assist in the modulation of inflammatory activation [22,43]. The kinase’s involvement in immune cell activity is primarily agonistic towards an inflammatory state, as its inhibition has been shown to negatively correlate with pro-inflammatory cytokine production and positively correlate with those that are anti-inflammatory in nature [57,80,81]. In addition to the modulatory role of GSK-3 in the realm of cellular differentiation, this supplementary ability to regulate the transcription and proliferation of various cytokines via the protein’s molecular interactions with the transcription factors NF-κB, STAT3, and many others, is paramount in solidifying the kinase’s involvement with the host immune response.

#### 3.3.1. Nuclear Factor Kappa B (NF-κB)

NF-κB is a transcriptional protein complex involved in the regulation and production of various cytokines and cell survival mechanisms [82]. The complex promotes pro-inflammatory states and is responsible for the production of IL-12, TNF-α, and IL-1β [22]. Additionally, NF-κB enhances the gene expression of inducible nitric oxide synthase (iNOS), which can promote oxidative stress in a given cell or system [83]. Importantly, GSK-3 has been shown in several studies to have a role in both the regulation of NF-κB and the inflammatory outcomes it promotes [57,84]. Indeed, the mechanism behind the kinase’s modulatory activity is multifactorial, as GSK-3 interacts with multiple proteins and complexes involved in the NF-κB transcriptional pathway [22,44].

The first of these interactions involves the stability of NF-κB with its functional subunits p50 and p65, which assemble to create the most common active form of NF-κB [82]. Notably, the precursor of the NF-κB p50 subunit, NF-κB p105, is phosphorylated and primed for degradation by GSK-3β when stimulated by TNF-α, giving rise to the NF-κB functional subunit p50 [85]. GSK-3β has a multitude of effects on the p65 subunit, as the kinase phosphorylates the protein at several sites, offering varying effects on NF-κB functionality between pro-inflammatory p65 ser536 phosphorylation and pro-survival p65 ser468 phosphorylation [86,87,88]. For example, the inhibition of *GSKβ* with TDZD-8 in rats significantly reduces p65 phosphorylation at ser536 in the spinal cord and lung during inflammatory states, such as spinal cord trauma and zymosan-induced non-septic shock, decreasing the pro-inflammatory activity of NF-κB, whereas the phosphorylation of p65 at ser468 by GSK-3 deactivates NF-κB when cells are unstimulated, maintaining basal levels of the factor [86,87,88]. Another aspect of NF-κB regulation by GSK-3 operates via the cellular concentration of β-catenin, a transcription factor directly controlled by the kinase through its retainment within the destruction complex [89]. β-catenin negatively regulates NF-κB in order to reduce bacteria-induced inflammation with both the inhibition of GSK-3 and the constitutively active expression of β-catenin, which is shown to reduce inflammatory responses in HCT116 CTNNB1 cell cultures exposed to bacteria with stabilized β-catenin [89]. 

Fundamental to the role of GSK-3 in the modulation of NF-κB and its inflammatory response is the binding of NF-κB to the transcriptional co-activator CBP (cAMP-response element binding protein (CREB)-binding protein) [57,90]. Importantly, NF-κB and the anti-inflammatory transcription factor CREB actively compete for the binding of CBP in order to initiate their respective effects [57,90,91]. Nuclear concentrations of CBP are finite and limited, resulting in CREB and NF-κB directly counteracting each other’s activation as they compete for the binding of the co-activator [57,90,91]. GSK-3 plays a significant role in this competition, as it inhibits CREB via the ser129 phosphorylation, inactivating the protein [92]. Importantly, the inhibition of GSK-3 has been shown to increase the nuclear levels of CREB with its subsequent binding to CBP, directly decreasing the expression of NF-κB [93]. Notably, like that of GSK-3, NF-κB activity can be stimulated by the activation of the TLR4, whereas CREB can be activated by the TLR2 through the PI3K-Akt-GSK-3 mediated pathway [57,58,91]. PI3K signaling is crucial for the activation of Akt, the kinase responsible for the deactivation of GSK-3 via ser9/21 phosphorylation [92]. Moreover, PI3K activates mTORC1, a protein complex responsible for the inhibition of GSK-3 via the disassociation between NF-κB and CBP [94,95]. Indeed, it has been reported that inhibition of PI3K resulted in the heightened production of numerous pro-inflammatory cytokines, such as IL-12, increased NF-κB p65 activity, and reduced IL-10 production [71,72,94]. Moreover, mice deficient in p85α, a regulatory subunit of the class IA PI3K, have demonstrated increased Th1-like immune responses during the immune challenge [71,72].

#### 3.3.2. Signal Transducer and Activation of Transcription (STAT)

Proteins that belong to the STAT family of transcription factors are responsible for the mediation and regulation of many cellular pathways, such as the host immune response, and receive activation from various cytokines that stimulate such processes [96]. The STAT family consists of numerous factors that collectively activate and promote the production of several inflammatory cytokines, such as IL-6, IL-1β, TNF-α, and IFN-inducible protein 10 [22,96,97]. Like that of NF-κB, the STAT family of transcription factors are subject to regulation by GSK-3 through a multitude of unique molecular pathways, with the most prominent GSK-3 actions on STAT1 and STAT3, which have been linked to the regulation of T cell differentiation [96]. The pro-inflammatory cytokine IFN-γ has been shown to promote the persistent activation of STAT1, with this interaction dependent on the IFN-γ receptor 2-associated Jak2 activation of GSK-3, which subsequently inhibits the src homology-2 domain-containing phosphatase 2 (SHP2)-mediated dephosphorylation of STAT1 [97]. Importantly, inhibition of the kinase has been shown to decrease the expression of STAT1-mediated proteins, such as TNFα, RANTES (chemokine ligand 5), and iNOS in RAW264.7 macrophages, along with a reduced IFN-γ production and cell count of Th1 in cultures of splenocytes from mice [97,98]. Such effects were mediated via the dephosphorylation of STAT1 by the Src homology-2 domain-containing phosphatase 2 (SHP2), which was augmented in response to GSK-3 inhibition [97].

With regard to STAT3, GSK-3 hosts the ability to modulate its activity in both a direct and indirect manner. Importantly, the cytokines IL-6 and IL-10, although opposite in inflammatory effect, both potentiate the function of STAT3 via time-dependent activation [99]. Specifically, upon stimulation of IL-10, which is actively inhibited by IFN-γ, STAT3 remains active for a prolonged period and has anti-inflammatory effects, whereas IL-6 promotes a transient effect that produces an opposing pro-inflammatory response [91,99]. The indirect method of regulation relies on GSK-3’s ability to attenuate the IL-10-induced phosphorylation of STAT3, preventing the expression of anti-inflammatory factors [91]. TLR2 stimulation has also been shown to promote STAT3-dependent expression and anti-inflammatory cytokine production [91]. The direct regulation of STAT3 by GSK-3 is dependent upon phosphorylation at the tyr705 site, which has been demonstrated by Beurel and Jope [100] in IFN-γ-stimulated murine astrocytes. Samavati and colleagues [101] discovered tyr705 phosphorylation of STAT3 to be necessary for the process of IL-1β and IL-6 production in LPS-stimulated cerebral cortical primary astrocytes, as the inactivation of GSK-3 led to a potent suppression of cytokine concentrations [101]. In another study by Beurel and colleagues [66], the pharmacological and molecular inhibition of GSK-3 blocked both IL-6 production and STAT3 activation, with the opposite effect observed with kinase stimulation. Complementary to such reports, the study of both GSK-3 isoforms under knockdown conditions demonstrated the importance of GSK-3β alone in the activity of STAT3 and STAT5, with GSK-3β knockdown resulting in both factors expressing a reduced functionality [100].

#### 3.3.3. Nuclear Factor-Erythroid Factor 2-Related Factor 2 (Nrf2)

Nrf2 is a cytoprotective transcription factor that hosts a critical role in the modulation of inflammation and intracellular oxidative stress [102]. The protein is responsible for inducing the expression of various detoxifying enzymes, such as heme oxygenase-1 (HO-1) and nicotinamide adenine dinucleotide phosphate (NADPH), along with numerous antioxidant genes [103]. Nrf2 actively inhibits the pro-inflammatory effects of NF-κB and has been shown to reduce inflammation via augmentation of the phagocytic removal of apoptotic cells and the downregulation of various cytokines, such as IL-1β, IL-6, and IL-17, with Nrf2 knockout increasing the susceptibility to pathogenic inflammation [104,105,106,107]. In recent years, Nrf2 has received increased attention amongst the scientific community for its potential as a therapeutic target in inflammatory disease. Most notably, the pharmacological activator of Nrf2, dimethyl fumarate (DMF) was recently approved as a therapeutic treatment for multiple sclerosis (MS), under the name of Tecfidera, and has been researched extensively, with the goal of potentially repurposing DMF for other inflammatory diseases, such as Parkinson’s disease [108]. Predictably, Nrf2, like that of NF-κB, CREB, and the STAT family of proteins, is subjected to regulation by GSK-3; however, unlike the aforementioned factors, Nrf2 is inhibited by the kinase.

GSK-3β directly phosphorylates Nrf2 at the ser335 and ser338 of its Neh6 domain, destabilizing the protein and allowing for the binding of β-transducin repeat-containing protein (β-TrCP), which marks the complex for proteasomal degradation, inhibiting its function as a cytoprotective factor against cellular stress [109,110]. Importantly, the pharmacological inhibition of GSK-3 by both TDZD-8 and lithium has been shown to increase Nrf2 expression in drosophila, as well as in mice with early-onset autosomal dominant polycystic kidney disease [111,112]. Additionally, the genetic knockdown of GSK-3 has shown similar results in drosophila [111]. PI3K and Akt, proteins involved in the regulation of GSK-3, have also been implicated in the regulation of Nrf2 as the pathway they promote the activity of Nrf2 through inhibition of GSK-3 [109,110,113]. Due to the actions GSK-3 has over the expression of Nrf2 target genes, current research has led to the development of novel multitargeted therapeutics that both inhibit GSK-3β and induce Nrf2 for the treatment of inflammatory diseases, such as Parkinson’s and Alzheimer’s disease [114,115].

## 4. GSK-3, Inflammation, and MDD

There is widespread evidence to suggest that chronic inflammation may have an important, if not causal role in the pathology of MDD ([12], reviewed in [116]). Importantly, the signaling of cytokines, key proteins in the host immune response, has been known to become dysregulated in MDD [117,118]. Specifically, elevated levels of pro-inflammatory cytokines, such as IL-12, IL-6, IL-1β, TNF-α, and IFN-γ, have all been documented in the plasma and serum of patients with MDD [119,120,121]. Additionally, several positive and negative correlations have been made between blood cytokine levels and the severity of depression. For example, the levels of some cytokines, such as IL-1β, TNF-α, IL-6, and IFN-γ, appear to correlate with the depressive symptom severity or the presence of suicidality based on Hamilton’s depression rating scale (HDRS), the Lethality Suicide Attempt Rating Scale (LSARS), and the Risk-Rescue Rating (RRR) [121,122]. Moreover, plasma IFN-γ and IL-4 ratios have been shown to be significantly increased in depressed individuals compared to controls, demonstrating an imbalanced immune response favoring a pro-inflammatory state [120]. Additionally, the popular antidepressant fluoxetine has demonstrated a modulatory effect over the host immune system via the attenuation of plasma and brain inflammatory cytokine levels, such as IL-1β, IL-6, and TNF-α, after 90 and 120-day treatment periods [123].

Key to the interplay between inflammation and MDD, IFN-γ, previously mentioned for its marked elevation in MDD, is known to inhibit the PI3K and Akt pathway and activate NF-κB, STAT3, and GSK-3, leading to a positive feedback loop, as Th1 and Th17 cells, both of which are stimulated by GSK-3, also induce the production of IFN-γ [46,73,91]. Of significance, it has been reported that mice deficient in p85α, a regulatory subunit of class IA PI3K, exhibit increased Th1-like immune responses during an immune challenge [71,72]. In this regard, the IFN-γ-induced activation of GSK-3 in MDD would further increase IFN-γ production via the NF-κB-mediated activation of Th1 and Th17 cells, leading to the increased suppression of PI3K (Figure 1).

In MDD, there is a tendency for T cell differentiation to elevate inflammatory Th1 and Th17 proliferation in tandem with Th2 and Treg cell downregulation [119,120,124,125,126]. The T cell modulatory cytokine, TGF-β, has had mixed reports, demonstrating both reduced [120,127,128] and increased [129] activity in patients with MDD. Interestingly, it has been postulated by Chen and colleagues [125], who found increased Th17 frequencies and decreased Treg concentrations in the blood of MDD patients, that very low concentrations of TGF-β may give rise to Th17 cells, whereas high concentrations of TGF-β may favor the production of Tregs. This inverse relationship is supported by additional clinical and preclinical studies that have shown an increased Th17:Treg ratio in MDD [124,125,126] and a downregulation of Th17 with a corresponding increase in Tregs in response to antidepressant administration in mice [130,131]. Additionally, the promotion of depressive-like behavior via the upregulation of Th1 and Th17 in a GSK-3 knockin mouse model has been shown, demonstrating the persistent activation of both GSK-3 isoforms [132]. In line with this evidence, the conflicting reports of TGF-β may correspond to the depressive susceptibility or advancement of the neuropathology.

Fundamental to the misbalanced differentiation of T cells, the cytokines responsible for the proliferation of Th1 and Th17 cells have been shown to be upregulated by GSK-3 via its regulation of inflammatory transcription factors, such as NF-κB, STAT3, and CREB, all of which have been implicated in rodent models of depression, as well as the activation of the TLR4 [43,50,66,133,134,135,136]. Interestingly, the upregulation of Nrf2 with the subsequent downregulation of TLR4/NF-κB signaling in the brain tissue of chronically mild stressed male mice has also been linked to the antidepressant-like effects of intravenously administered adipose-derived mesenchymal stem cells, as well as with the induction of brain-derived neurotrophic factor (BDNF) and tyrosine receptor kinase B (TrkB) expression [137,138].

Often critical to the development of MDD is psychological stress, most notably in the form of chronic mild stressors [139]. Psychological stress, such as inescapable foot shocks, social defeat stress, restraint stress, and social isolation, has been shown in multiple rodent models to induce both depression-like behavior and the activation of GSK-3 and NF-κB [39,51,52,53,57,59,137,140], which is likely due to the DAMP-mediated activation of the TLR4 [58,61,62,141]. Cheng and colleagues [58] demonstrated that the gene knockout of hippocampal TLR4 in male mice exposed to inescapable foot shocks resulted in the absence of stress-induced hippocampal GSK-3 and NF-κB activation, concomitant with the absence of a depressive-like phenotype. Interestingly, the effect of TLR4 knockout to prevent stress-induced NF-κB activation was mimicked by the antidepressants, fluoxetine, and the GSK-3 inhibitor TDZD-8 [58]. In MDD, GSK-3 upregulation may augment the activity of NF-κB and subsequently downregulate the activity of CREB. This phenomenon was alluded to in the study of the effects of the antidepressant baicalin by Yu et al. [142], in which hippocampal and hypothalamic levels of IL-1β, IL-6, and TNF-α in male rats were found to be negatively regulated by the drug through the NF-κB pathway.

Taken together, these findings, in combination with the investigations demonstrating GSK-3 inhibitors as potent anti-inflammatory and anti-depressant agents, demonstrate the potential strong overlap between the activity of GSK-3 in both the host immune response and pathology of MDD [57,95]. We posit that this may indicate that GSK-3 acts as a point of convergence for the etiology of feed-forward pathogenic inflammation in MDD, as has been suggested for neurodegenerative disorders, such as Parkinson’s and Alzheimer’s disease [17].

## 5. System-Wide Effects of Pathogenic GSK-3 and Inflammation in MDD

Fundamental to the role of GSK-3 in MDD is the extensive downstream effects that both the kinase and the host immune system disrupt in order to further the pathology (Figure 1). GSK-3 acts as a key regulator in the immune system, and also acts on the host of other systems, such as the 5-HT, glutamatergic, and cholinergic neurotransmission signaling systems. Having a critical link between the many networks of the human brain, GSK-3 may act as a convergence point that promotes its ongoing activity in a feed-forward manner through these signaling networks, introducing a system-wide disruption in neuronal functionality and individual well-being.

### 5.1. The Serotonin Inhibition by Indoleamine 2,3-Dioxygenase (IDO)

Reduced 5-HT neurotransmission is perhaps the most well-known and widely studied hallmark of MDD [143]. Various mechanisms have been shown to be linked to reduced 5-HT signaling in the disorder, such as gene mutations in the 5-HT transporter (SERT) [144,145] or 5HT-1A receptor [146,147], as well as reduced 5-HT synthesis [148]. Interestingly, STAT3 has been critically linked to the IL-6-dependent inhibition of 5-HT signaling [133]. It was discovered by Kong et al. [133] that IL-6 reduced SERT activity, mRNA, and protein levels in JAR cells and hippocampal neurons in vitro. Intracerebroventricular administration of IL-6 was also shown to reduce hippocampal SERT expression [133]. Consistent with these findings, IL-6-knockout (KO) mice exhibited elevated SERT expression and displayed a reduction in depression-like behavior and decreased sensitivity to acute antidepressant treatment [133]. Additionally, STAT3 was found to associate with the SERT promoter in an IL-6-dependent manner, with the inhibition of STAT3 blocking the effect of IL-6 on 5-HT uptake in vitro and reducing depression-like behavior in vivo [133]. Fundamental to these findings, as they directly contradict the anti-depressant effects of SERT antagonism by SSRIs, is the discovery of both a reduced SERT function in several rodent models of depression [149,150] and evidence demonstrating that the knockout of the transporter induced depressive behavior in mice [151].

Mounting evidence suggests that a major mechanism underlying the marked reduction in 5-HT activity [148,152] in MDD is the suppression of tryptophan (TRP) metabolism resulting from the upregulation of IDO during inflammatory stress (reviewed in [153,154,155]). IDO, the enzyme responsible for the oxygenation of TRP into kynurenine (KYN) in extrahepatic tissue, has a ubiquitous presence throughout the human body and can be found within various immune cells, such as macrophages and microglia [156]. In line with this, a reduction in serum IDO levels appears to be correlated with antidepressant-induced symptom improvement in humans, with the activation of IDO shown to promote depression-like behavior in mice [154,157]. The elevated production of inflammatory cytokines directly activates IDO, further reducing 5-HT levels in the brain and body [156,158,159,160]. Along with TNF-α, IL-6, and IL-1β, which can directly activate IDO via NF-κB, another prominent cytokine in MDD responsible for the activation of IDO within macrophages and microglia is IFN-γ [158,159,160]. Notably, IFN-γ has the most potent effect on the IDO activation of any cytokine and has been shown to be inhibited by multiple antidepressants, with a subsequent increase in anti-inflammatory IL-10 production functioning in order to inhibit IDO and normalize TRP concentrations [153,161,162]. Another enzyme, known as tryptophan 2,3-dioxygenase (TDO), also reduces 5-HT concentrations in the brain via KYN metabolism; however, TDO is only present in the liver, having an effect only on TRP circulating throughout the body rather than directly in the brain [163,164]. TDO can be activated by glucocorticoids, such as cortisol, introducing another positive feedback loop for the 5-HT reduction in MDD [153].

#### 5.1.1. IDO Metabolites

IDO creates two main metabolites from KYN once TRP has been oxygenated: quinolinic acid (QUIN) and kynurenic acid (KYNA) [153,156]. QUIN, a potent glutamatergic NMDA agonist, is increased in the CSF of those that have attempted suicide compared to healthy controls [165], and functions to induce toxic oxidative and nitrosative stress, contributing to further inflammation and cell trauma, as well as astrocytic and neuronal death [166,167]. The mechanism by which QUIN induces oxidative and nitrosative stress is two-fold, one process acting via the activation of NMDA receptors, discussed in more detail later in the review, and the second process acting via the modification of endogenous antioxidant enzyme activity in order to prevent cytosolic free radical scavengers, such as cytosolic copper/zinc superoxide dismutase (CuZn-SOD), from clearing toxic metabolites [153,168,169]. Both pathways induce a cytosolic increase in free radicals, such as reactive oxygen species (ROS) and nitric oxide (NO), with the subsequent induction of nitric oxide synthase (NOS) [167,168,169]. Importantly, oxidative stress can trigger the activation of NF-κB, which can subsequently initiate the production of iNOS, as well as various inflammatory cytokines, leading to the progression of MDD and heightened GSK-3 activity [170]. Interestingly, TRP has been shown to induce the activation of Nrf2, an inducer of the antioxidant pathway [171]; however, with the metabolism of TRP by IDO, such concentrations are reduced, suppressing Nrf2 activity. Elevated serum levels of iNOS have been reported in individuals with mild to severe depression, with significantly increased mRNA expression of iNOS encoding genes in the peripheral blood cells of depressed individuals as well [172,173]. Additionally, iNOS levels show marked increases in hippocampal microglia following chronic unpredictable mild stress models of depression in mice [174]. GSK-3 inhibitors have been shown to significantly reduce iNOS concentrations in various studies of microglial cells and macrophages, with STAT1-mediated iNOS production dependent on GSK-3 [44,49,78,97]. Furthermore, noradrenaline reuptake inhibitors (NRIs), which inhibit GSK-3 [175], have been shown to greatly reduce the gene expression of iNOS both in vitro in cultured glial cells and in vivo in rats in order to induce anti-depressant effects [176].

The other metabolite produced by IDO, KYNA, hosts a different role in the etiology of MDD, one that largely affects the cognitive ailments of the condition. KYNA, mainly produced in astrocytes, is an antagonist of the α7 nicotinic acetylcholine receptor (α7nAChR) [177], a receptor implicated in cognitive processes such as learning and memory. Specifically, the selective activation of the receptor improved the cognitive ability in rodent model tests of object recognition memory and attenuated age-related spatial learning deficits [177,178]. GSK-3 has been linked to α7nAChR activation, as the receptor stimulates the activation of the PI3K/Akt/GSK-3β pathway as part of the cholinergic anti-inflammatory response [179,180,181]. Agonists for the receptor are being investigated for therapeutic use in human disease; however, unfavorable side effects and pharmacokinetic issues have prevented the development of effective clinical α7nAChR agonists [182,183,184]. Recent evidence suggests that the inhibition of hippocampal KYNA may represent an alternative and effective strategy for cognitive enhancement [185], as seen in various rodent models [176,185].

#### 5.1.2. Crosstalk with Cholinergic Signaling and Oscillatory Dysfunction

Fundamental to system-wide cognitive processes is the regulation of neuronal oscillatory activity, a mechanism in which the α7nAChR plays a key role [186,187]. Neuronal oscillations are synchronous, macroscopic electrical rhythm (brain wave)-generated populations of neurons that allow for the functional system-wide communication of the nervous system [188]. In cognitive processes, such as learning and memory, both low-frequency theta oscillations and high-frequency gamma oscillations in the ventral hippocampus (HIP) and various cortical regions are critical [188,189,190,191]. Importantly, it has been shown that the selective activation of α7nAChR can modulate HIP oscillations, specifically by enhancing theta power and theta phase–gamma amplitude coupling and promote tetanically induced gamma waves in rat HIP slices [186,187]. With the antagonism of the α7nAChR by KYNA, these processes are disrupted, and potentially contribute to the various cognitive and oscillatory impairments noted in MDD [192]. The mechanism by which α7nAChR regulates neuronal oscillations is unknown; however, the activation of the receptor has been shown to inhibit GSK-3 through a PI3K-Akt pathway, as mentioned previously [179,180,192]. Notably, it has been reported by Albeely et al. [188] that increasing GSK-3 activity in rat PFC or HIP can influence neuronal oscillations both within and between both regions. Additionally, Nguyen et al. [193] demonstrated that the inhibition of GSK-3 in rats, by a selective inhibitor or lithium, also influenced both theta and gamma band activity. GSK-3β inhibition in the corticolimbic interneurons of Ppp1r2-cre/floxed-Grin1 knockout mice has also been shown to both ameliorate deficits in spatial working memory and evoke gamma power increases in the primary auditory cortex [194]. Importantly, evidence suggests that reductions in 5-HT signaling, the main outcome of IDO’s metabolic process, may also influence neuronal system activity. As reported by Xu et al. [195] using male rats, the activation of the 5-HT1A receptor strengthened HIP ventral CA1–PFC coupling in gamma oscillations and weakened HIP CA1 theta–fast gamma cross frequency coupling, establishing the 5-HT system as having a key role in the regulation of oscillatory coupling in the HIP vCA1–mPFC network [195]. Although more research is required, it is apparent that a critical link exists between α7nAChR, neuronal oscillations, oxidative stress, and GSK-3, which may provide further insight into the pathological feed-forward activation of GSK-3 in MDD.

#### 5.1.3. Serotonin Regulation of Host Immune System

Along with IDO metabolite toxicity, as alluded to previously, reduced 5-HT signaling in MDD can lead to elevated GSK-3 activity and the expedited progression of inflammatory insult. It has been delineated that some 5-HT receptors, such as the 5-HT2A receptor, inhibit GSK-3 activity, linking 5-HT to the feed-forward mechanism of pathogenic GSK-3-induced inflammation directly, as the reduced 5-HT levels noted in MDD would remove this additional form of inhibition over GSK-3 [35,196]. Additionally, 5-HT has been shown to modulate various cells of the adaptive immune system. Specifically, 5-HT can act directly on Th1 and Th17 cells in order to reduce the production of IFN-γ and IL-17, as demonstrated in MS patients by Sacramento et al. [197]. The study also provided evidence indicating 5-HT as having the ability to enhance Treg function and IL-10 secretion by naïve T cells, further reducing inflammation [197]. Notably, the activation of the 5-HT2B receptor inhibited the differentiation of human moDC-primed CD4+ T cells into Th1 effector lymphocytes in inflammatory settings [198]. 5-HT regulation of Th2 is poorly understood (reviewed in [199]), although it has been recognized that a lack of 5-HT in allergic airway inflammation has the capacity to impair Th2 priming in dendritic cells [200]. Kant and colleagues [201] also established that IL-6 produced by Th17-stimulated microglia in encephalomyelitis (EAE) mice inhibited the synthesis of 5-HT via the reduction in tetrahydrobiopterin, which happened in synergy with the TNF-α activated IDO cycle, further reducing 5-HT in the brain. Furthermore, Kong and colleagues [133], as mentioned in the prior section, confirmed the further involvement of IL-6 and 5-HT via the identification of the IL-6/STAT3-dependent reduction in SERT in JAR cells, which was ameliorated in IL-6-knockout mice HIP tissue. With regard to Tregs, deficiencies in 5-HT synthesis and signaling via TRP hydroxylase knockout and 5-HT2A receptor antagonism have displayed phenotypic reductions in the immune cells in mice, primarily due to a mechanism of the 5-HT2A receptor activation on CD4+ T non-Tregs, promoting Treg proliferation [201,202]. Overall, 5-HT, when physiologically normal, will promote the activation of Tregs and possibly Th2 cells with the subsequent inhibition of Th1 and Th17 cells, augmenting self-tolerance and inhibiting immune stress [203]. In MDD, when 5-HT levels are systematically low, this process would promote the reverse scenario, favoring chronic inflammation and the feed-forward mechanism posed by this review. Taken together, these interactions between GSK-3, IDO, and the adaptive immune system, in combination with the resulting suppression of 5-HT signaling, establish a complex relationship of associated pathways that advance the neuropathological cycle of MDD.

#### 5.1.4. Inflammatory Disruption of Other Monoaminergic Signaling Pathways in MDD

Other monoamines, such as DA and norepinephrine (NE), have also been linked to inflammatory processes [12] and GSK-3 signaling [204,205,206]. Yan et al. [207] established the ability of DA to inhibit systemic inflammation through the inactivation of inflammasomes via D1R signaling. Furthermore, IFN-α treated rhesus monkeys exhibit depressive-like behavior, concomitant with decreased DA release in the striatum, as well as lower CSF concentrations of the DA metabolites homovanillic acid (HVA) and 3,4-dihydroxy-phenylacetic acid (DOPAC) [208,209]. Moreover, the DA reuptake inhibitor, bupropion, has anti-inflammatory effects with the subsequent suppression of TNF-α synthesis via the mediation of increased β-adrenoreceptor and D1R signaling [210]. In addition to DA, NE signaling reductions in MDD have been a well-documented phenomenon for several years, with various antidepressants acting as inhibitors of NE reuptake (reviewed in [211,212]). Notably, NE hosts a regulatory role in the host immune response, as the neurotransmitter has been shown to reduce cortical rat microglial IL-1β and NOS productions, with noradrenergic neuron denervation significantly increasing astrocytic activity during TNF-α-mediated activation [213,214]. Interestingly, the inhibitory neurotransmitter GABA, also reduced in MDD, has the ability to modulate the immune system, as it both ameliorates EAE and is itself inhibited by chronic inflammation via IL-1β, as shown in MS patients [13,215,216]. Importantly, DA [204], NE [205,206], and GABA [217] independently act to inhibit GSK-3 activity, with their depletion in MDD suggesting a systemic disinhibition of the kinase in the pathology.

### 5.2. Glutamatergic Dysregulation

In contrast to the reductions in 5-HT observed in MDD, GLU signaling and, in particular, signaling through the NMDA receptor, is increased, resulting in a number of pathogenic consequences, including excitotoxicity and oxidative stress [10]. Aberrant NMDA activity in MDD involves two major pathways that are directly linked to GSK-3, furthering the neuronal insult. Extensively covered in the 5-HT section, the first and most notable loop is through IDO’s metabolite, QUIN, a substrate of nicotinamide adenine dinucleotide (NAD) [153]. Through the elevated production of QUIN during inflammatory stress, a heightened NMDA activation causes a substantial influx of calcium ions and the activation of NOS, resulting in the increased production of NO and damaging free radicals, such as ROS, and leading to states of oxidative stress [167]. As a result of such a cellular strain, NF-κB activity is elevated, leaving the opportunity for inflammation-mediated GSK-3 stimulation. Additionally, NMDA channel activation has been shown to activate GSK-3 directly [218]. Demonstrated by Szatmari et al. [218], NR2B-subunit-containing NMDA channels cause the protein phosphatase 1 (PP1)-mediated disinhibition of GSK-3β in the cultured hippocampal and cortical neurons of rats. Moreover, this initiates an active GSK-3 positive feed-back loop as GSK-3β activated PP1 as well, further enhancing the activation of the phosphatase, additively inhibiting CREB via GSK-3 [218]. A second feed-forward loop operates through GSK-3 itself, as the kinase has been shown to prevent the internalization of NR2B-subunit-containing NMDA receptors, as demonstrated by Chen et al. [219] through the use of GSK-3 inhibitors, which promoted receptor sequestration in cortical pyramidal neuron cultures. Notably, Yang and colleagues [220] also found that the pharmacological agent Kukoamine provided neuroprotective effects in cultured primary cortical neurons via the downregulation of NR2B-containing NMDA receptors and the subsequent phosphorylation of Akt and GSK-3β. In this regard, elevated NMDA signaling, both through enhanced activation and NR2B channel concentrations, would promote an increase in GSK-3 activation, further augmented by the PP1 function.

In addition to the NMDA-mediated induction of oxidative stress and GSK-3 activation, aberrant NMDA receptor activity has been implicated in dysfunctional BDNF signaling, which is notably reduced in MDD [221]. BDNF activates PI3K, Akt, and mTORC1 via its receptor TrkB and is neuroprotective [221,222,223]. Interestingly, mTORC1 activation, via its upstream regulator sestrin, has shown strong antidepressant effects, as demonstrated by Kato et al. [224], normalizing BDNF signaling deficits in male rats that were exposed to chronic unpredictable stress. It is believed that excessive NMDA stimulation in MDD results in the inhibition of BDNF synthesis via the phosphorylation of eukaryotic elongation factor 2 kinase (EEF2K), an inducer of BDNF transcription [225]. In addition, α-amino-3-hydroxy-5-methyl-4-isoxazolepropionic acid (AMPA) signaling has been shown to enhance BDNF levels and is believed to play a major role in the anti-depressant effects of ketamine [225,226]. Importantly, in rodent models of MDD, the NMDA/AMPA ratio is increased, with AMPA stimulation reduced due to NMDA oversaturation [227]. Ketamine effectively increases the AMPA/NMDA ratio and increases BDNF and mTOR signaling, providing fast acting anti-depressant effects [223,228]. BDNF signaling inhibition, as would be seen in MDD, would result in increased GSK-3 activity via the deactivation of Akt and mTORC1, an effect augmented by the NMDA receptor’s additional ability to reduce Akt activity [229]. The antidepressant fluoxetine has also been shown to increase Akt activity, which subsequently deactivates GSK-3 in a phosphorylation-dependent manner [41]. One additional NMDA receptor-related pathway of MDD pathogenesis involves the relationship between NMDA receptor activation and pro-inflammatory cytokine activity. Recently, it was reported by Francija et al. [230] that NR2A knockout mice had elevated proBDNF in the PFC and HIP, resilience to depressive-like behavior upon LPS stimulation, and a lack of an inflammatory response. Additionally, it has been reported that IL-1β has the ability to increase NMDA receptor activity in primary HIP neuron cultures via the activation of tyrosine kinases and subsequent NR2A/B subunit phosphorylation [231]. Thus, with the increase in pro-inflammatory cytokines in MDD, this would contribute to another mechanism to promote hypophosphorylation and the increased activation of GSK-3. Lastly, for the receptor, it is well-documented that NMDA plays a key role in the regulation of neuronal oscillations [231,232]. It is postulated that elevated NMDA activation results in a reduced gamma power observed in MDD, as NMDA receptor ablation results in heightened gamma activity and, in line with this idea, ketamine has also been shown to increase the gamma power [232,233,234,235]. Taken together, these findings collectively highlight GSK-3 as a possible central convergence point for aberrant GLU signaling in the context of chronic inflammation in MDD.

### 5.3. Aberrant Insulin Signaling in MDD

Insulin and the closely related insulin growth factor-1 (IGF-1) and IGF-2 ligands mediate a variety of biological functions, such as the promotion of growth through two highly related tyrosine kinase receptors—the insulin receptor and the IGF-1 receptor, both highly expressed throughout the human brain [236]. Insulin resistance reduces cell and receptor sensitivity to insulin signaling, along with its biological effects. In recent years, insulin resistance has become a well-established hallmark of MDD, as reports have indicated that insulin resistance, MDD, and inflammatory stress are correlated [11]. Like that of BDNF, insulin can suppress GSK-3 activity via the activation of Akt, as well as protein kinase C (PKC) and protein kinase B (PKB) [17,236]. GSK-3 suppresses insulin receptor substrate 1 (IRS-1) activation, with GSK-3 inhibition resulting in a reduced insulin resistance and augmented insulin functionality [237,238]. In MDD, the emergence of insulin resistance results in a number of downstream consequences that allow for the subsequent upregulation of both GSK-3 and inflammatory processes.

Insulin signaling was shown to augment BDNF transport deficits, increase BDNF mRNA, phosphorylate Akt, inhibit GSK-3, and reduce depressive-like behavior in the FST of mice [239,240]. The pharmacological sensitization of insulin by dicholine succinate (DS) in chronically stressed C57BL6 mice reduced depressive and anxiety-like behaviors and normalized the expression of the NMDA NR2A subunit in the HIP, along with the balancing of NR2A/NR2B subunit ratios [241]. Additionally, insulin has been found to play a key role in the regulation of 5-HT and DA through several modulatory mechanisms (reviewed in detail in [242]). Briefly, insulin has the ability to enhance the quantity of TRP entering the brain, which has been established by the administration of insulin that enhanced 5-HT synthesis in the HIP of diabetic mice [243,244]. Moreover, insulin reduces the function of the monoamine-degrading enzyme A (MAO-A) and MAO-B, which subsequently metabolizes and reduces synaptic 5-HT and DA [245].

## 6. Conclusions

Chronic inflammation and elevated GSK-3 activity are two major hallmarks of MDD. In this review, it has been hypothesized that these two processes are intimately connected and, indeed, function as a feed-forward mechanism to advance MDD pathogenesis. Specifically, with an initial insult of stress-meditated activation of the TLR4, it is possible that GSK-3 actions, in concert with inflammatory cytokines, such as IFN-γ and TNF-α, promote the development of MDD via the reduction in 5-HT and insulin signaling, upregulated NMDA transmission, and aberrant neuronal oscillatory functionality. In addition to MDD onset, these factors have been shown to work together to effectively impair the recovery process of learned helplessness models of depression via the inflammatory disruption of the blood–brain barrier, contributing to the long-term occurrence of the condition [246]. Thus far, clinical and preclinical work have failed to find a safe treatment for MDD that shows rapid therapeutic efficacy, the notable exception being the novel antidepressant ketamine, which has its own concerns surrounding its potential side effects.

The use of monotherapeutic strategies such as SSRIs has been the mainstay of MDD intervention; however, the neuropathology of MDD is multi-faceted, involving numerous processes that may be exploited, potentially through a combined therapeutic approach. This is also worth exploring in the context of treatment-resistant depression, where standard therapies are ineffective. One possibility is through the use of GSK-3 inhibitors as therapeutic agents, as this protein kinase appears centrally linked to many of the pathogenic pathways linked to MDD.

## Data Availability

Not applicable.
